# Evaluation of the impact of an online video game as an educational intervention on sexual health and the prevention, diagnosis, and treatment of sexually transmitted infection: A randomized controlled trial protocol

**DOI:** 10.1186/s12909-024-05903-3

**Published:** 2024-08-26

**Authors:** Alba Martinez-Satorres, Carme Roca-Saumell, Anna Escale-Besa, Marta Arcarons-Marti, Francisco Javier Fernandez-Segura, Carolina Allegra Wagner, Pablo Pires-Nuñez, Nuria Turmo-Tristan, Lorena Diez-Garcia, Andrea Maron-Lopez, Zulema Marti-Oltra, Marta Vanrell-Nicolau, Sonia Da Silva Torres, Alvaro Ruiz-Torres, Pablo Pino-Prieto, Dhyaanenshan Pillay, Angels Casaldaliga-Sola, Xavi Lazaro-Navarro, Maria Lasagabaster-Uriarte, Maria Isabel Fernandez-San Martin

**Affiliations:** 1 Gerència d’Atenció Primària Barcelona Ciutat, Institut Català de La Salut (ICS), Carrer Balmes, Barcelona, 22, 08007 Spain; 2 Grup de Dermatologia de La Societat Catalana de Medicina Familiar I Comunitària (CAMFiC), Carrer Diputació, Barcelona, 316, 08009 Spain; 3 Unitat Docent Multiprofessional d’Atenció Familiar I Comunitària de Barcelona Ciutat, Barcelona, Spain; 4 Facultat de Medicina I Ciències de La Salut, Universitat de Barcelona (UB), Carrer Casanova, Barcelona, 143, 08036 Spain; 5 Gerència d’Atenció Primària Catalunya Central, Institut Català de La Salut (ICS), Carrer Pica d’Estats, 13-15, Sant Fruitós de Bages, Barcelona, 08272 Spain; 6 Gerència d’Atenció Primària Metropolitana Sud, Institut Català de La Salut (ICS), Carrer Balmes, Barcelona, 22, 08007 Spain; 7 Grup de Violències Masclistes de La Societat Catalana de Medicina Familiar I Comunitària (CAMFiC), Carrer Diputació, Barcelona, 316, 08009 Spain

**Keywords:** Sexually transmitted infections, Sex education, Health education, Health promotion, Sexual health, Gamification

## Abstract

**Background:**

The incidence of sexually transmitted infections (STIs) is increasing, especially among young people. Tools are needed to increase knowledge about sex education and STI prevention and treatment. Gamification can be a good training tool for both young people and health professionals. The primary objective of this study is to assess the impact of a training intervention on STI prevention, detection, and treatment in primary care professionals.

**Methods/design:**

Multicentre cluster randomized controlled trial.

Groups of primary care professionals will receive an intervention (online video game on sex education and STIs [SEXIT]) and will be compared with control groups that will not receive the intervention. Group assignments will be randomized by clusters.

The study will consist of a pre-post evaluation of the intervention: a knowledge test will be administered before and after the intervention and 3 months after the intervention. This test will also be carried out on the same time sequence in the control groups. The impact of the training intervention will be assessed over a 6-month period, focusing on various variables associated with the clinical management of STIs. This evaluation entails the clinical records of diagnostic tests and antibiotic prescriptions related to the clinical approach to STIs.

The required sample size is 262 (131 per group).

**Discussion:**

Compared with those in the control group, improvements in knowledge and clinical behavioural outcomes after the intervention are expected for participants in the intervention groups. We plan to develop an educational video game to increase the knowledge about sexuality, STIs and violence.

Protocol registered at ISRCTN with reference number **ISRCTN17783607**.

## Background

Over the last decade, there has been a clear overall increase in the incidence of *Chlamydia trachomatis*, gonorrhoea, syphilis, and L. venereum in Europe (Área (Área [1]); , Centro (Centro [2])). According to a study by the Barcelona STI and HIV Group published in 2019 (Sentís et al. (Sentís et al. [3])), between 2007 and 2015, the incidence of STIs significantly increased among young people aged 15–24 years, and the importance of improving programmes and interventions targeting STIs in young people is stressed. The group also found that a history of a previously diagnosed STI, being a man who has sex with men and having a greater number of sexual partners are risk factors for HIV coinfection in young people with gonorrhoea, syphilis, or lymphogranuloma; these are therefore the people targeted for screening and educational interventions.

Intimate partner violence (IPV) is associated with several high-risk sexual behaviors, including inconsistent condom use, multiple sexual partners, early sexual debut, substance use during sexual activity, and a higher prevalence of sexually transmitted infections (Seth [4]); , Stubbs and Szoeke (Stubbs and Szoeke [5])). Female IPV victims exhibit higher rates of STIs and engage in more STI-risk behaviors compared to women in non-violent relationships. Therefore, women in violent relationships should be prioritized for STI screening in clinics. Additionally, STI prevention messages should address IPV issues due to their significant impact on STI risk (Hess et al. (Hess et al. [6])).

A questionnaire for the detection of male violence was carried out on 1,566 young women aged 15 to 33 years who were users of the Youth Centre for Sexuality Care [7] between April 2017 and January 2019. According to the data extracted from the answers to this questionnaire, 5.2 out of every 10 women surveyed had suffered at least one situation of physical, psychological, or sexual violence.

The aim of this project is to design, develop and evaluate an educational intervention aimed at residents and primary care professionals to improve people’s sexual health and the prevention, detection, and treatment of STIs.

### Justification

Among the possible causes of difficulty in dealing correctly with STIs may be a lack of specific training from professionals, a lack of knowledge about safer practices among the most vulnerable population, and a barrier to accessing the health system.

In this context, teaching tools that encourage active participation in learning are needed. New teaching methods have been used whereby students learn without being directly taught. Based on more participatory methods, it is not the teacher who provides the student with the information, but rather the student who learns thanks to the teaching dynamic. Gamification is an example of this. Gamification is a relatively new trend that involves applying game mechanics to nongame contexts to engage audiences, generate fun, and produce motivational and cognitive benefits. In the field of education, gamification is a formative process through which learning experiences are seen as games. It is currently one of the most attractive methodologies and has aroused great interest, topping the list of new teaching methods in terms of its effectiveness (Beemer et al. (Beemer et al. [8]); , Grimalt (Grimalt [9])).

### Theoretical framework for learning with computer games for education

Transfer involves applying what you have learned in one context to solve problems or learn in a new context. Multimedia learning scenarios incorporate both words (printed or spoken) and pictures (graphics, animation, and video). Mayer’s Cognitive Theory of Multimedia Learning (CTML) explains how learning occurs in these multimedia situations, based on three key principles (Mayer and Mayer (Mayer and Mayer [10]))

1. Dual Channel Principle: People have separate channels for processing visual and verbal material.
2. Limited Capacity Principle: Each channel can process only a limited amount of material at one time.
3. Active Processing Principle: Deep learning occurs when people actively engage in cognitive processing during learning.

**Fig. 1:**
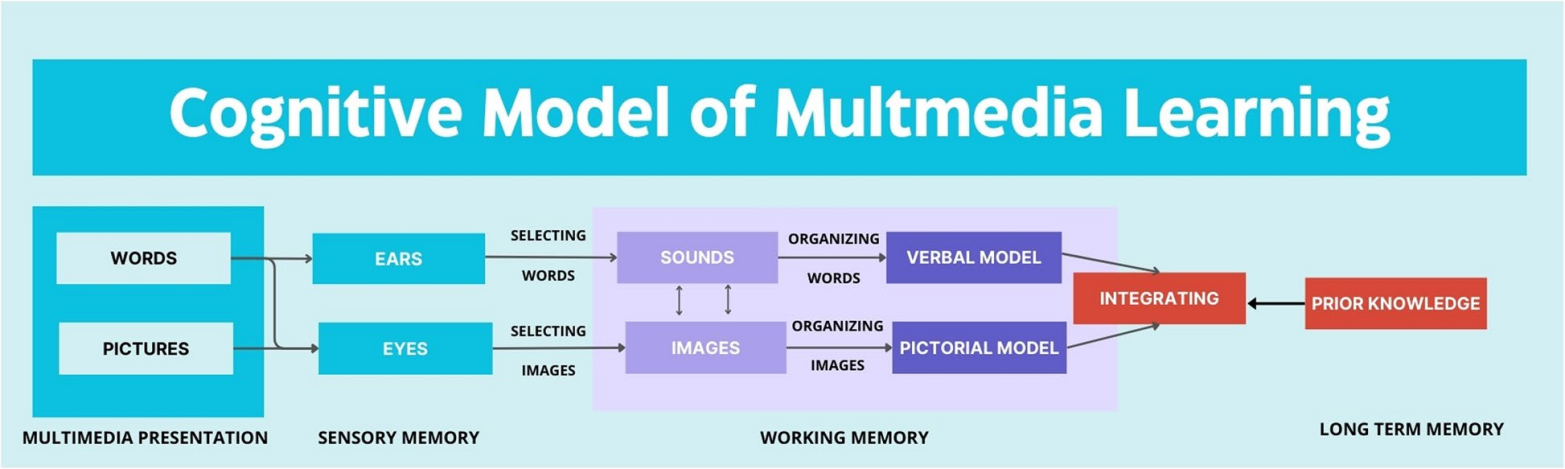
Mayer’s Cognitive Model of Multimedia Learning: this model summarizes the cognitive processes and mental representations involved in multimedia learning

By integrating these principles (Fig. 1), gamified training interventions can create more engaging, effective, and efficient learning experiences that align with the cognitive processes of learners. CTML principles help ensure that gamified training aligns with how people process information, enhancing learning outcomes.

A 2023 article meta-analysed 21 studies that tested the effectiveness of animated videos in improving learning in clinical and nonclinical settings compared with standard education.

Mayer’s Cognitive Theory of Multimedia Learning provided the theoretical model to frame the current analyses. Findings indicated an overall positive effect (d = 0.35) for use of animation in improving viewers’ learning across a variety of health and clinical contexts (Feeley et al. (Feeley et al. [11])).

Gamification strategies include the serious game, in which learning takes place through an organized game with a set of rules and an objective. This type of game creates a challenge, involves interaction, and has a theme or thread. It is devised specifically to promote health and, at the same time, to be fun (DeSmet et al. (DeSmet et al. [12])), promoting group integration and cohesion. Advances in technology allow these strategies to be used when face-to-face activities may not be effective.

The team game allows the application of debriefing (Maestre et al. (Maestre et al. [13])) methodology, involving a conversation to review a simulated event in which players analyse their actions and reflect on the reasoning, skills, and emotional states generated in the simulated situation to improve or maintain their performance in the future. Decisions and mistakes are reflected upon together. It helps participants not only increase their knowledge but also change their attitudes and practices by providing questions based on reflection on their own mistakes and offering opportunities for learning, reflection, and attitudinal changes.

A review of 30 studies with 3,634 participants concluded that gamification appears to be at least as effective as traditional formal teaching (Gentry et al. (Gentry et al. [14])). Moreover, it appears to be even more effective at improving learning, skills, and satisfaction. More rigorous studies of higher quality are needed to assess whether gamification can lead to real learning more effectively than traditional teaching.

Another systematic literature review was conducted to examine gamification strategies in e-Health, assessing their benefits and challenges. A total of 46 studies were thoroughly analyzed. The review found strong evidence that gamification aids cognitive development, enhancing strategic abilities, working memory, visual attention, and processing speed. Despite challenges, most studies highlighted the positive effects of gamified e-Health interventions and serious games, making typically mundane activities more enjoyable and engaging. Gamification also improved user experiences and provided extrinsic motivation and positive emotional states.

(Sardi et al. (Sardi et al. [15])). A third review of 40 studies of educational interventions using gamification with healthcare professionals also concluded that it is possible to improve learning outcomes in health profession education through gamification, especially when using game attributes that improve learning behaviours and attitudes towards learning (Van Gaalen et al. (Van Gaalen et al. [16])). High satisfaction rates and positive changes in behaviour and learning have been reported.

However, many studies had short evaluation periods, reducing result accuracy. Therefore, long-term empirical evaluations are recommended for gamified applications, especially in therapy and prevention.

Furthermore many of the reviewed studies do not compare the results with equivalent control groups, so there is a need to delve deeper into and explore theories that can explain the effects of gamified interventions with well-defined longitudinal control groups (Manterola et al. (Manterola et al. [17])). All three reviews agree that additional studies are needed in this regard.

Kirkpatrick developed an organizational model that has been used for the evaluation of training actions (Johnston et al. (Johnston et al. [18]); –Pertiñez (Pertiñez [20])). It is based on the classification of learning on four levels:

1. Reaction: Participants’ perceptions or satisfaction with training interventions immediately after receiving them.
2. Learning: knowledge and skills acquired by taking, for example, a knowledge test before and after the intervention.
3. Attitude or application of learning: the application of knowledge in the workplace and, consequently, any changes in service delivery. It is recommended to wait at least 3–6 weeks to evaluate this phenomenon.
4. Outcomes: assesses whether the learning is transferred to the clinical setting and whether it improves patient outcomes. This could be the impact of the training on the population.

Educational impact assessment provides valuable information for educators to assist in the development and improvement of teaching methods. In training activities, it is important not only to assess the impact on knowledge gain but also to determine whether this learning translates into changes in attitudes and clinical practices after the intervention (Norman (Norman [21])). To date, online learning is known to be at least as effective as traditional learning in terms of learning acquisition, but studies evaluating the third and fourth levels, i.e., the impact of educational interventions on changing practitioner attitudes and improving patient goals, are still scarce (DeSilets et al. (DeSilets et al. [22]); , Sinclair et al. (Sinclair et al. [23])).

### Hypothesis

A gamified educational intervention, the SEXIT videogame, will generate knowledge about sexuality education; access to health care; and the prevention, diagnosis, and treatment of sexually transmitted infections. It will contribute to better health care and promote better sexual health at the community level.

### Main objective

The purpose of this study is to evaluate the impact of a training intervention in the form of an online video game aimed at improving sexual health and STI prevention, detection, and treatment in primary care professionals.

### Specific objectives

- To assess the intervention’s impact on knowledge about sexual and reproductive health.
- To assess and detect behaviours and knowledge for the prevention of gender violence and/or violence based on sexual identity or orientation.
- To describe the changes in the clinical management of STIs: screening and diagnostic tests performed, diagnoses and antibiotic prescriptions.

## Methods

We will design, develop, and evaluate an educational intervention in the form of a video game aimed at primary care professionals. The intervention will be studied and compared with control groups that will not carry out the intervention.

Design: Design-cluster randomized clinical trial with pre-post evaluation. The PCC teams (health professionals working in a primary care centre (doctors, nurses and residents)) will be randomly assigned to the intervention or control group.

The intervention groups are doing a preintervention test, which will be repeated immediately after the intervention and then 3 months later. In the control groups, the test will be administered at the beginning of the study and repeated after 3 months. In addition to questions to assess knowledge, there will be a qualitative assessment satisfaction survey for the participants. This test will also be carried out at the same time in control groups with the same sociodemographic characteristics.

The intervention will consist of an online video game developed by a multidisciplinary team.

## Study population, site participation, and recruitment

The study will be conducted in primary care centres (PCCs) managed by the Institut Català de la Salut (ICS, Catalan Health Institute), the main primary care service provider in Catalonia, with the participation of family and community medicine areas and nursing residents and professionals from PCCs of the public health system.

The study will start on April 2024 (Timeline in Table 1).

**Table 1:** Timeline

Task	Date
Questionnaires design and piloting	2023
Recruitment of PCC	January—May 2024
Training Intervention	May–June 2024
Data collection from questionnaires	May – October
Clinical data collection	May – December

### Recruitment of participants

**Fig. 2:**
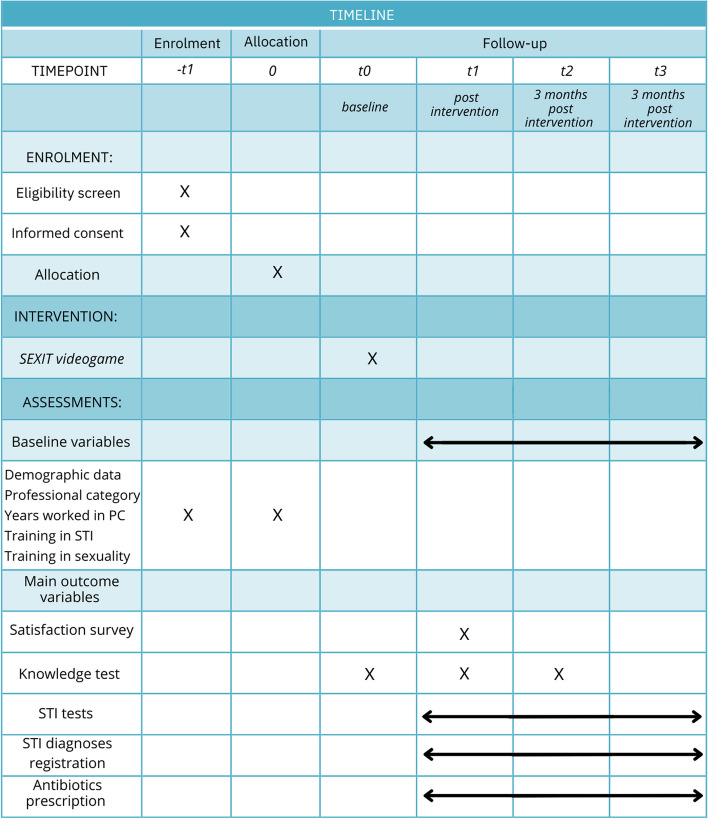
Timeline schedule

The PCCs (Table 2) will be invited to participate in the study by training referents. Participation in the study will be proposed by the primary care training referral platform. A letter and a slide presentation will be made to explain the study. Once the PCCs who wish to participate have been selected, they will be randomly assigned to a control/intervention group (Fig. 2).

**Table 2:** PCC invited to participate in the study

Territorial management of primary care	Primary care center
Barcelona	CAP Adrià
	CAP Besòs
	CAP Bon Pastor
	CAP Bordeta-Magòria
	CAP Casc Antic*
	CAP Casernes
	CAP Chafarinas
	CAP Ciutat Meridiana
	CAP Doctor Carles Ribas
	CAP Doctor Lluís Sayé*
	CAP Drassanes*
	CAP El Carmel
	CAP El Clot*
	CAP Encants*
	CAP Gòtic*
	CAP Guinardó
	CAP Guineueta
	CAP Horta
	CAP La Marina
	CAP La Mina
	CAP La Pau*
	CAP La Sagrera
	CAP Les Indianes
	CAP Manso
	CAP Montcada i Reixac*
	CAP Montnegre
	CAP Numància
	CAP Pare Claret
	CAP Passeig de Sant Joan*
	CAP Poblenou
	CAP Ramon Turró
	CAP Rio de Janeiro
	CAP Roger
	CAP Roquetes-Canteres
	CAP Sanllehy
	CAP Sant Andreu
	CAP Sant Martí de Provençals
	CAP Sant Rafael
	CAP Sants
	CAP Trinitat Vella
	CAP Turó
	CAP Vila de Gràcia-Cibeles
Catalunya Central	CAP Navàs Balsareny*
	CAP Sant Joan de Vilatorrada*
Metropolitana Nord	CAP Mollet del Vallès Oest*
Metropolitana Sud	CAP Vilavella (St Vicenç dels Horts—Torrelles) *

Centres marked with an asterisk are already enrolled

### Assignment of intervention/control groups

The assignment will be randomized by clusters (PCC).

Once the centres have been recruited, they will be stratified and matched according to the following variables: teaching/nonteaching status, classification according to the MEDEA index, percentage of assigned population of migrant origin and number of family doctors and primary care nurses with assigned quotas.

Centres with similar characteristics will be randomly assigned to the control or intervention group. The allocation of PCCs to each group will be made by a person outside the circle of researchers using a table of random numbers.

The individuals in the control group will be able to carry out the training activity once the study will be completed.

## Evaluation outcomes

### Independent variables

Educational intervention: The training activity will consist of a video game accessed online from a computer and played in teams of 4–6 people. The game consists of an online escape room where the resolution of various chained tests allows knowledge to be acquired.

### Variables that may act as confounders or effect modifiers

- Demographic data: gender (male/female/nonbinary), sexual orientation (heterosexual/homosexual/bisexual/asexual/don’t want to answer), and age.
- Professional category (medicine resident/nursing resident/doctor/nurse).
- Years worked in primary care.
- Training experience in STI
- Training experience in sexuality.
- PCC.
- Variables of the PCC: teaching/nonteaching; classification according to MEDEA; percentage of assigned population of migrant origin; number of family doctors and primary care nurses with assigned quota.

### Main outcome

The impact of the intervention will be assessed at three levels following Kirkpatrick’s model: reaction, learning, and clinical behaviour change.

### Dependent variables

1. **Reaction**: assessment of satisfaction with the intervention. Satisfaction will be measured with a qualitative survey on the formative activity that will be administered to the intervention groups after playing the game (Table 3).
2. **Learning **

**Table 3:** Satisfaction survey

**Question**	Answer
Level of satisfaction with the activity	Likert 1–5
The format of the activity allows learning to be achieved	Likert 1–5
Satisfaction with the downloadable materials	Likert 1–5
Do you think it will change your attitude towards addressing sexuality/STIs?	Likert 1–5
Positive aspects to highlight	Open
Negative aspects or suggestions	Open

Knowledge change will be measured with a self-developed questionnaire. Prior to implementation, the questionnaire will be validated through evaluation by a group of health professionals with expertise in STIs and pilot testing by resident doctors and nurses who will not subsequently participate in the study. The clarity of the questions, comprehension of the instructions, length of the test, and relevance of the distractors will be evaluated. Modifications suggested in the expert judgement and pilot testing will be incorporated into the questionnaire.

The questionnaires will include:

Knowledge test (Table 4): 25 multiple choice questions with 4 possible answers.

**Table 4:** Knowledge test

Area	Questions
Prevention and detection of gender violence	1, 2, 3, 4, 5
Emergency contraception and abortion	6, 7
Anamnesis	8
Screening and diagnosis	9, 10, 11
Transmission	12, 13, 14
STI approach and treatment	15, 16, 17, 18, 19, 20, 21, 22, 23, 24, 25
Extra question – no points	26

The items included in the questionnaire will be prevention of gender violence and/or violence based on sexual identity or orientation and clinical approach to STI.

3. Evaluation of changes in the application of the knowledge acquired in clinical practice

The impact of the training intervention will be evaluated for 6 months by studying different variables related to the clinical approach to treating STIs:

- Performance of diagnostic tests: multitest PCR, exudate culture, and serology.
- Recording of aetiologically oriented health problems in clinical history.
- Number of epidemiological surveys (data provided by the Public Health Agency).
- Prescription of antibiotics.

These clinical data will be collected from health professionals during the 6 months following the intervention, both in the intervention and control groups.

Antibiotic use data will be collected from electronic prescriptions generated by health professionals.

## Data collection and sources of information

The participants in each group will complete an initial knowledge test. Once the first knowledge test will be completed, the participants in the intervention group will carry out the training activity, and at the end of the activity, they will answer the knowledge test again and complete a satisfaction survey on the same platform. The control group will not complete these post-intervention questionnaires. After 3 months, the knowledge test will be repeated for both the intervention and control groups. The data will be collected and stored in the same way.

Clinical data (request for tests, recording of health problems and antibiotic prescriptions) will be extracted from the computerized medical records.

Data from epidemiological surveys of notifiable diseases will be requested from the Public Health Agency.

### Study population

*Inclusion criteria for participants* will be as follows: family and community medicine and nursing residents; family doctors; and primary care nurses with assigned quotas.

*Exclusion criteria* will be: not having online gaming devices; not being able to follow up for 6 months; being an STI referral professional (STI referrers are those professionals who, after specific training, are designated as consultants with or without their own STI agenda).

## Statistical analysis

### Calculation of sample size

The sample size was determined based on findings from a pilot test involving pre- and post-intervention assessments conducted on 35 health professionals. Using the mean intervention effect (the mean test score improvement) and its standard deviation to estimate the expected intervention effect and within-group variability in the pilot test, the mean improvement was 2.07, with a standard deviation of 4.07. Assuming an alpha error of 0.05, a beta error of 0.2, and a design effect factor of 2 and expecting a 20% loss to follow-up after a 6-month period, the required sample size would be 262 (131 per group).In addition, the proportion of the LGTBIQ + population was considered to carry out a subsequent analysis from a gender and LGTBIQ + perspective. To estimate the proportion of LGTBIQ + professionals, data from the IPSOS (DeSilets et al. (DeSilets et al. [22])) survey were used; 14% of the Spanish population is estimated to belong to this group. This is the same proportion obtained in the sample of the pilot test (5 individuals (14.29% of the 35 participants) identified as LGTBIQ + ..

### Planned analysis

For the treatment and analysis of the data, a descriptive analysis of the variables (percentages and averages with measures of dispersion) will be carried out according to the nature of the variables.

1. Demographic and background data: descriptive statistics will be used (mean, median, standard deviation, range) to summarize the characteristics of the participants, including age, sex, years worked, etc. The distribution of test scores will be used to observe the distribution of pre- and postintervention test scores.
2. Sensitivity analysis of the instrument: Analyses will be repeated using different methods to verify the robustness of the results.
3. Comparison of baselines – Mann‒Whitney U or independent sample T-tests: whether the groups will be comparable in terms of demographic characteristics and baseline scores. Chi-square tests will be used to compare categorical proportions between groups (if applicable).
4. Analysis of the effect of the intervention: T-tests for dependent samples or Wilcoxon signed-rank tests will be used to compare pre- and postintervention values within the same group. ANCOVA (analysis of covariance): to control for confounding or baseline variables, ANCOVA could be useful. Mixed analysis of variance (ANOVA): for multiple repeated measures (for example, pre, post- and 3-month follow-up).
5. Cluster analysis (if applicable): Multilevel analysis or mixed models. These models will be useful for considering the hierarchical nature of clusters in the data.
6. Lost data management – sensitivity analysis: To determine if missing data affects the results. Imputation techniques such as multiple imputation could be used if there is a significant amount of missing data.

### Limitations

The intervention to be assessed cannot be masked since it could influence its effect. Because there is a possibility of contamination bias among professionals, randomization by clusters is proposed under the assumption that the number of clusters would be sufficient for each group.

Geographic dispersion of the PCC will reduce the possibility of contamination.

Participants will know they are being assessed, which could lead to observer bias, although comparisons with a control group who will also know they are being observed may reduce the effect of this bias in the surveys. On the other hand, regarding the clinical variables of the professionals, it is thought that the observation time is long enough that the fact that they are being observed does not interfere with their clinical use.

## Discussion

We expect to obtain a validated educational tool that, through gamification, allows primary care professionals to increase their knowledge of sexuality, sexually transmitted infections, and the prevention and detection of violence, improving the results of the evaluation test after the educational intervention.

Expected results:

- An increase in the knowledge of sexuality, sexually transmitted infections, and the prevention and detection of violence among primary care professionals, as well as an improvement in the results of the evaluation test after the educational intervention and improved results in the participants of the intervention groups compared to their control groups.
- An increase in the number of diagnostic and screening tests performed after the intervention in comparison to the control group.
- A better recording of diagnoses and more appropriate antibiotic treatments for the groups of professionals for whom the intervention will be carried out.

The aim of this study is to evaluate the tool used by primary care professionals But in the near future it is expected to be possible to play the game at different levels of difficulty.. The level of difficulty will depend on the videogame players: professionals, university students of health sciences or patient populations as young people.

We expect to build an educational tool that motivates active participation and facilitates acquisition of knowledge and safe behaviours related to sexual health in the short and medium term, including the prevention and detection of violence based on gender, sexual identity or orientation, access to the health system, and prevention, detection, and treatment of sexually transmitted infections. Likewise, we expect this tool to be a facilitator of health education for at-risk or vulnerable groups.

## Data Availability

The datasets used and/or analysed during the current study are will be available from the corresponding author on reasonable request.

## Abbreviations

LGTBIQ +: Lesbian, gay, bisexual, trans, intersex, queer and plus
PC: Primary care
PCC: Primary care centre
PCR: Polymerase chain reaction
RCT: Randomized controlled trial
STI: Sexually transmitted infections
